# An institutional perspective on the impact of recent antibiotic exposure on length of stay and hospital costs for patients with gram-negative sepsis

**DOI:** 10.1186/1471-2334-12-56

**Published:** 2012-03-13

**Authors:** Scott Micek, Michael T Johnson, Richard Reichley, Marin H Kollef

**Affiliations:** 1 Pharmacy Department, Barnes-Jewish Hospital, St. Louis, Missouri; 2 Department of Pharmacy Practice, UIC-College of Pharmacy, Chicago, IL, USA; 3 Hospital Informatics Group, BJC Healthcare, St. Louis, Missouri; 4 Pulmonary and Critical Care Division, Washington University School of Medicine, St. Louis, Missouri; 5 Division of Pulmonary and Critical Care Medicine, Washington University School of Medicine, 660 South Euclid Avenue, Campus Box 8052, St. Louis MO 63110, USA

**Keywords:** Antibiotics, Severe sepsis, Outcomes, Length of stay, Hospital costs

## Abstract

**Background:**

Prior antibiotic exposure has been associated with the emergence of antibiotic resistance in subsequent bacterial infections, whose outcomes are typically worse than similar infections with more antibiotic susceptible infections. The influence of prior antibiotic exposure on hospital length of stay (LOS) and costs in patients with severe sepsis or septic shock attributed to Gram-negative bacteremia has not been previously examined.

**Methods:**

A retrospective cohort study of hospitalized patients (January 2002-December 2007) was performed at Barnes-Jewish Hospital, a 1200-bed urban teaching hospital. Patients with Gram-negative bacteremia complicated by severe sepsis or septic shock had data abstraction from computerized medical records. We examined a consecutive cohort of 754 subjects (mean age 59.3 ± 16.3 yrs, mean APACHE II 23.7 ± 6.7).

**Results:**

*Escherichia coli *(30.8%), *Klebsiella pneumoniae *(23.2%), and *Pseudomonas aeruginosa *(17.6%) were the most common organisms isolated from blood cultures. 310 patients (41.1%) had exposure to antimicrobial agents in the previous 90 days. Patients with recent antibiotic exposure had greater inappropriate initial antimicrobial therapy (45.4% v. 21.2%; p < 0.001) and hospital mortality (51.3% v. 34.0%; p < 0.001) compared to patients without recent antibiotic exposure. The unadjusted median LOS (25^th ^percentile, 75^th ^percentile) following sepsis onset in patients with prior antimicrobial exposure was 13.0 days (5.0 days, 24.0 days) compared to 8.0 days (5.0 days, 14.0 days) in those without prior antimicrobial exposure (p < 0.001). In a Cox model controlling for multiple confounders, prior antibiotic exposure independently correlated with remaining hospitalized (Adjusted hazard ratio: 1.473, 95% CI: 1.297-1.672, p < 0.001). Adjusting for potential confounders indicated that prior antibiotic exposure independently increased median attributable LOS by 5.0 days. Similarly, total hospital costs following sepsis onset was significantly greater among patients with prior antimicrobial exposure (median values: $94,737 v. $21,329; p < 0.001).

**Conclusions:**

Recent antibiotic exposure is associated with increased LOS and hospital costs in Gram-negative bacteremia complicated by severe sepsis or septic shock. Clinicians and hospital administrators should consider the potential impact of recent antibiotic exposure when formulating empiric treatment decisions for patients with serious infections attributed to Gram-negative bacteria.

## Background

Knowledge of the clinical and economic impact of antimicrobial resistance is useful to influence programs and behavior in healthcare facilities, to guide policy makers and funding agencies, to define the prognosis of individual patients and to stimulate interest in developing new antimicrobial agents and therapies [1]. One of the most misunderstood issues is how to measure cost appropriately when analyzing the antibiotic treatment of infections. Although imperfect, existing data show that there is an association between antimicrobial resistance in *Staphylococcus aureus*, enterococci and Gram-negative bacilli and increases in mortality, morbidity, length of hospitalization and cost of healthcare [1]. Patients with infections due to antimicrobial-resistant organisms have greater hospital costs (US$6000-30,000) than do patients with infections due to antimicrobial-susceptible organisms [1,2]. Given limited budgets, imperfect knowledge of the clinical and economic impact of antibiotic-resistant bacterial infections, coupled with the benefits of specific interventions targeted to reduce these infections or to improve their treatment, more optimal management strategies to control and improve antibiotic utilization are needed. The traditional way of accounting for antimicrobial costs in terms of pharmacy acquisition expenditures for antibiotics may not be appropriate when discussing the treatment of antibiotic resistant infections. Instead, increasing evidence suggests that the most cost-effective approach for the treatment of such infections is to provide appropriate therapy in a timely manner [3-6].

Inappropriate initial antimicrobial therapy, defined as an antimicrobial regimen that lacks in vitro activity against the isolated organism(s) responsible for the infection, has been associated with excess mortality and lengths of stay in patients with severe sepsis and septic shock [5,7-11]. This is largely related to increasing bacterial resistance to antibiotics as a result of their greater use and limited availability of new agents [12,13]. Escalating rates of antimicrobial resistance lead many clinicians to empirically treat critically ill patients with presumed infection with a combination of broad-spectrum antibiotics, which can perpetuate the cycle of increasing resistance [14]. Conversely, inappropriate initial antimicrobial therapy can lead to treatment failures and adverse patient outcomes [15]. Therefore, clinicians need to be aware of when it is clinically and economically reasonable to use newer, broad-spectrum antibiotics. Prior antibiotic exposure may represent an important marker for decision making, especially as new broad-spectrum antibiotics begin to enter the clinical setting.

The most recent Surviving Sepsis Guidelines recommend empiric combination therapy targeting Gram-negative bacteria, particularly for patients with known or suspected *Pseudomonas *infections, as a means to decrease the likelihood of administering inappropriate initial antimicrobial therapy [16]. The choice of an antimicrobial regimen that is active against the causative pathogen(s) is increasingly problematic as the treating physician usually does not know the susceptibilities of the pathogen(s) for the selected empiric antibiotics at the time of antibiotic prescription. We previously showed that recent antibiotic exposure is associated with increased hospital mortality in Gram-negative bacteremia complicated by severe sepsis or septic shock, primarily due to inappropriate initial treatment [17]. Therefore, we performed an investigation to determine whether recent antibiotic exposure also resulted in increased LOS and hospital costs among patients with severe sepsis or septic shock attributed to Gram-negative bacteremia.

## Methods

### Study Location and Patient Population

This study was conducted at a university-affiliated, urban teaching hospital: Barnes-Jewish Hospital (1200 beds). Over a six-year period (January 2002-December 2007) all hospitalized patients with Gram-negative bacteremia were eligible for inclusion. This study was approved by the Washington University School of Medicine Human Studies Committee. Barnes-Jewish Hospital is part of BJC HealthCare, one of the largest nonprofit health care organizations in the United States, delivering services to residents primarily in the greater St. Louis, southern Illinois and mid-Missouri regions. BJC serves urban, suburban and rural communities and includes 13 hospitals and multiple community health locations which share a common informatics system for medical records.

### Study Design and Data Collection

Utilizing a retrospective cohort study design, patients with Gram-negative sepsis were identified by the presence of a blood culture positive only for Gram-negative bacteria combined with primary or secondary ICD-9-CM codes indicative of acute organ dysfunction. Patient specific baseline characteristics and process of care variables were collected from the automated hospital medical record, microbiology database, and pharmacy database of Barnes-Jewish Hospital. Data collection for all patients was uniform regardless of the initial location of their hospitalization (intensive care unit or general hospital ward). Only the first episode of severe sepsis or septic shock attributed to Gram-negative bacteremia was evaluated. Electronic inpatient and outpatient medical records available for all patients in the BJC Healthcare system were reviewed to determine prior antibiotic exposure.

The baseline characteristics collected included: age, gender, race, past history of congestive heart failure, chronic obstructive pulmonary disease, diabetes mellitus, chronic liver disease, underlying malignancy, and end-stage renal disease requiring dialysis. The Acute Physiology and Chronic Health Evaluation (APACHE) II [18] and Charlson co-morbidity scores were calculated based on clinical data present during the twenty-four hours after the positive blood cultures were obtained. This was done to accommodate patients with community-acquired and healthcare-associated community-onset infections who only had clinical data available after blood cultures were drawn. The primary outcome variable was total hospital LOS. Secondary outcomes evaluated included: total hospital costs and proportion of patients receiving inappropriate initial antimicrobial therapy.

### Definitions

All definitions were prospectively selected prior to initiation of the study. LOS after onset of severe sepsis or septic shock was defined as the number of hospital days following the drawing of the first positive blood culture in eligible patients. To be included in the analysis patients had to meet criteria for severe sepsis based on discharge ICD-9-CM codes for acute organ dysfunction as previously described [19]. The organs of interest included the heart, lungs, kidneys, bone marrow (hematologic), brain, and liver. Patients were classified as having septic shock if vasopressors (norepinephrine, dopamine, epinephrine, phenylephrine or vasopressin) were initiated within 24 hours of the blood culture collection date and time.

Antimicrobial treatment was classified as appropriate if the initially prescribed antibiotic regimen was active against the identified pathogen based on in vitro susceptibility testing and administered within 24 hours following blood culture collection. For patients with polymicrobial infection the initial antimicrobial regimen had to be active against all identified pathogens in order to be classified as appropriate. Appropriate antimicrobial treatment also had to be prescribed for at least 24 hours. However, the total duration of antimicrobial therapy was at the discretion of the treating physicians. Prior antibiotic exposure was any exposure to an antibiotic within the preceding 90 days.

Total hospital costs following sepsis onset were derived from a summation of costs incurred across hospital cost centers, including room and board, pharmacy, radiology, and laboratory. Cost data were directly reported from the Barnes-Jewish Hospital accounting department; cost:charge ratios were not used. All reported costs represent actual costs for the administration of patient care as determined by the individual departmental finance sections.

### Antimicrobial Monitoring

From January 2002 through the present, Barnes-Jewish Hospital utilized an antibiotic control program to help guide antimicrobial therapy. During this time the use of cefepime and gentamicin were unrestricted. However, initiation of intravenous ciprofloxacin, imipenem, meropenem, or piperacillin/tazobactam was restricted and required pre-authorization from either a clinical pharmacist or infectious diseases physician. Each intensive care unit had a clinical pharmacist who reviewed all antibiotic orders to insure that dosing and interval of antibiotic administration was adequate for individual patients based on body size, renal function, and the resuscitation status of the patient. After daytime hours the on call clinical pharmacist reviewed and approved the antibiotic orders. The initial antibiotic dosages employed for the treatment of Gram-negative infections at Barnes-Jewish Hospital were as follows: cefepime, 1 to 2 grams every eight hours; pipercillin-tazobactam, 4.5 grams every six hours; imipenem 0.5 grams every six hours; meropenem, 1 gram every eight hours; ciprofloxacin, 400 mg every eight hours; gentamicin, 5 mg/kg once daily.

### Antimicrobial Susceptibility Testing

The microbiology laboratory performed antimicrobial susceptibility of the Gram-negative blood isolates using the disk diffusion method according to guidelines and breakpoints established by the Clinical Laboratory and Standards Institute (CLSI) and published during the inclusive years of the study [20,21].

### Data Analysis

Categorical variables were compared with the Fisher's exact test. We compared normally distributed continuous variables via the Student's *t*-test while employing the Mann-Whitney *U *test for non-parametric, continuous factors. We constructed curves for LOS after onset of severe sepsis or septic shock according to the methods of Kaplan and Meier to examine time to hospital discharge as a function of prior antibiotic exposure. These curves were compared via log-rank test. All tests were two tailed and unpaired while, a p value of < 0.05 was assumed to represent statistical significance.

To evaluate the independent impact of prior antibiotic exposure on hospital LOS, we created a Cox-proportional hazards model. Prior to completing the model we determined that the proportional hazards assumption was not violated. A priori, we placed in this model factors we felt that were biologically likely to affect hospital LOS. Specifically, we controlled for age, gender, co-morbidities, infection source, severity of illness, pre-infection hospital LOS (for nosocomial infections), inappropriate antibiotic therapy, prior hospitalization, and hospital-onset infection. Variables were examined to assess for co-linearity. We then re-calculated the Kaplan-Meier survival curves for the probability of remaining hospitalized controlling for each independent factor which remained significant in the Cox model but plotted two separate curves - one for patients without prior antibiotic exposure and another for those with prior antibiotic exposure. From this we determined the median adjusted difference in hospital LOS between the two groups. The correlation between LOS and hospital costs was assessed using Spearman's rank correlation coefficient. All analyses were completed using SPSS 11.0 (SPSS, Chicago, IL).

## Results

Seven hundred fifty-four consecutive patients with bacteremic Gram-negative severe sepsis or septic shock were included in the study. The mean age was 59.3 ± 16.3 years (range, 18-99 years) with 394 (52.3%) males and 360 (47.7%) females (Table 1). There were 421 (55.8%) medical patients and 333 (44.2%) surgical patients. The mean duration of hospitalization was 10.2 ± 14.4 days (range, 1-96) at the time severe sepsis or septic shock occurred. The mean APACHE II score was 23.7 ± 6.7 (range, 4-43) with the majority of patients requiring vasopressors (58.5%) and mechanical ventilation (55.3%). *Escherichia coli *(30.8%), *Klebsiella pneumoniae *(23.2%), *Pseudomonas aeruginosa *(17.6%), *Enterobacter *species (10.1%), *Acinetobacter *species (8.4%), *Proteus mirabilis *(4.9%), *Serratia marcescens *(4.0%), and *Stenotrophomonas maltophilia *(1.9%) were the most common organisms isolated from blood cultures. Fifty-six (7.4%) patients had polymicrobial bacteremia.

**Table 1:** Baseline Characteristics*

Variable	Prior Antibiotic Exposure (n = 310)	No Prior Antibiotic Exposure (n = 444)	P- value
Age (years)	56.9 ± 16.6	60.9 ± 16.0	0.001

Male	170 (54.8)	224 (50.5)	0.235

Infection Onset Type			

Community-acquired	0 (0.0)	71 (16.0)	<0.001

Healthcare-associated community-onset	30 (9.7)	236 (53.2)	

Healthcare-associated hospital-onset	280 (90.3)	137 (30.9)	

Duration of hospitalization prior to sepsis (days)	20.4 ± 17.1	3.2 ± 5.3	<0.001

Underlying Comorbidities			

Congestive Heart Failure	53 (17.1)	91 (20.5)	0.243

Chronic Obstructive Lung Disease	62 (20.0)	74 (16.7)	0.241

Chronic Kidney Disease	39 (12.6)	70 (15.8)	0.221

Liver Disease	37 (11.9)	57 (12.8)	0.712

Active Malignancy	95 (30.6)	146 (32.9)	0.517

Diabetes	65 (21.0)	104 (23.4)	0.426

APACHE II score	23.5 ± 6.5	23.8 ± 6.9	0.525

Charlson co-morbidity score	4.3 ± 3.7	5.2 ± 3.6	0.002

In ICU when sepsis occurred	265 (85.5)	331 (74.5)	<0.001

Vasopressors	207 (66.8)	234 (52.7)	<0.001

Mechanical Ventilation	221 (71.3)	196 (44.1)	<0.001

Drotrecogin alfa (activated)	8 (2.6)	23 (5.2)	0.077

Organ Dysfunction			

Cardiovascular	214 (69.0)	253 (57.0)	0.001

Respiratory	238 (76.8)	228 (51.4)	<0.001

Renal	163 (52.6)	240 (54.1)	0.690

Hepatic	24 (7.7)	31 (7.0)	0.693

Hematologic	88 (28.3)	141 (31.8)	0.322

Neurologic	17 (5.5)	31 (7.0)	0.407

Number of organ failures	2.4 ± 1.0	2.1 ± 1.1	<0.001

Source of Bacteremia^#^			

Lungs	166 (53.5)	134 (30.2)	<0.001

Urinary Tract	67 (21.6)	163 (36.7)	<0.001

Central Venous Catheter	16 (5.2)	40 (9.0)	0.047

Intra-abdominal	64 (20.6)	73 (16.4)	0.141

Unknown	8 (2.6)	41 (9.2)	<0.001

Values are expressed as number (%) and mean ± standard deviation.

APACHE = acute physiology and chronic health evaluation; ICU = intensive care unit.

*Data from hospital admission (demographics and underlying comorbidities) or within 24 hours of obtaining a positive blood culture in patients with severe sepsis or septic shock.

^#^Defined using Centers for Disease Control criteria (www.cdc.gov/ncidod/dhqp/pdf/nnis/NosInfDefinitions.pdf)

Three hundred-ten (41.1%) patients had prior antibiotic exposure during the preceding 90 days. Of these, cefepime was the most common agent with previous exposure (50.0%) followed by ciprofloxacin (32.6%), imipenem or meropenem (28.7%), pipercillin-tazobactam (19.0%), and aminoglycosides (14.5%). Most prior antibiotic exposure was during the same hospitalization and within 21 days of severe sepsis onset for patients with healthcare-associated hospital-onset infections (77.9%). Among patients with healthcare-associated community-onset infections prior antibiotic exposure was either outpatient administered (56.7%) or administered during a hospitalization occurring within 90 days of infection onset (43.3%). When compared to cases with no prior antibiotic exposure, patients with prior exposure were significantly more likely to have healthcare-associated hospital-onset sepsis, sepsis occur in the intensive care unit setting, and a longer duration of stay prior to sepsis onset (Table 1). Patients with prior antibiotic exposure were also significantly younger, had lower Charlson co-morbidity scores, were more likely to have a pulmonary source of infection, and to require mechanical ventilation and vasopressor support.

Patients with prior antibiotic exposure had higher rates of inappropriate initial antimicrobial therapy (45.5% v. 21.2% p < 0.001) and hospital mortality (51.3% v. 34.0%, p < 0.001) compared to patients without prior antibiotic exposure. Inappropriate antibiotic therapy of severe sepsis was more likely to be associated with prior exposure to cefepime (37.4% v.12.9%; p < 0.001), ciprofloxacin (25.1% v. 8.1%; p < 0.001), ceftriaxone (7.2% v. 1.3%; p < 0.001), and imipenem (13.6% v. 5.4%; p < 0.001) compared to appropriate therapy. Similarly, prior exposure to cefepime, ciprofloxacin, and imipenem were associated with statistically longer hospital LOS.

**Figure 1:**
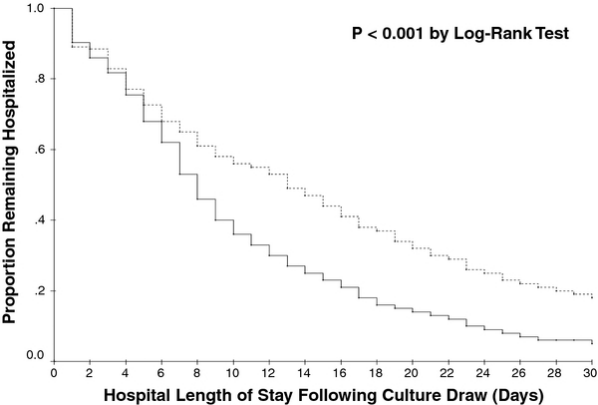
**Kaplan Meier curves showing the proportion of patients remaining hospitalized after the onset of severe sepsis or septic shock attributed to Gram-negative bacteremia**. The solid line represents patients without prior antibiotic exposure and the broken line represents patients with prior antibiotic exposure. P < 0.001 by Log-Rank test.

Figure 1 reveals the probability of remaining hospitalized after the diagnosis of severe sepsis or septic shock as a function of prior antibiotic exposure. Subjects receiving prior antibiotic exposure had an unadjusted median increase of 5.0 days in LOS (13.0 days v. 8.0 days, p < 0.001). In a Cox model adjusting for multiple co-variates (see Table 2), prior antibiotic exposure remained linked with an increased probability of remaining hospitalized after the onset of Gram-negative sepsis. The adjusted hazard ratio related to prior antibiotic exposure equaled 1.473 (95% CI: 1.297-1.672). Other variables significantly correlated with remaining hospitalized included pre-infection length of stay, severity of illness, inappropriate antibiotic therapy, hospital-onset infection, and a pulmonary source of infection. After controlling for the other factors displayed in Table 2, we estimate that prior antibiotic exposure independently increased hospital length of stay by approximately 5.0 days.

**Table 2:** Independent Factors Associated With Length of Stay

Variable	Hazard Ratio (95% CI)	*P *Value
Prior antibiotic exposure	1.473 (1.297-1.672)	0.002

Pulmonary source of infection	1.670 (1.496-1.865)	<0.001

APACHE II Score (1-point increments)	1.066 (1.057-1.076)	<0.001

Inappropriate antibiotic therapy	1.390 (1.239-1.559)	0.004

Hospital-onset infection	1.486 (1.234-1.790)	0.034

Pre-culture length of stay (1-day increments)	1.012 (0.008-1.016)	0.001

CI = confidence interval; APACHE = Acute Physiology and Chronic Health Evaluation

Other variables included in the analysis for which the Cox-proportional hazards model was adjusted included male gender, age (1-year increments), underlying liver disease, underlying renal disease, underlying malignancy, prior hospitalization (within 90 days), and Charlson co-morbidity score. Hazard ratios greater than 1 indicate the factor is associated with a greater probability of remaining hospitalized. Patients who died were censored at the time of death because they did not reach the endpoint of "discharged alive from hospital."

**Figure 2:**
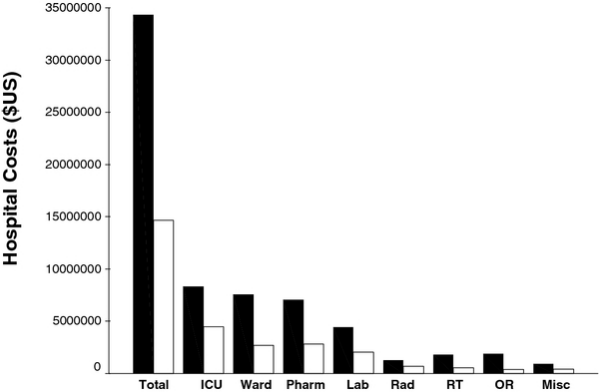
**Bar graph depicting total hospital costs and individual cost centers costs**. Black bars represent patients with prior antibiotic exposure and white bars represent patients without prior antibiotic exposure. ICU = intensive care unit, Pharm = pharmacy, Lab = laboratory, RT = respiratory therapy, OR = operating room, RAD = radiology, Misc = miscellaneous.

Figure 2 depicts the hospital costs following the onset of Gram-negative sepsis for the main cost centers examined. Total median hospital costs were significantly greater for patients with prior antibiotic exposure compared to patients without prior antibiotic exposure (median values: $94,737 [25^th ^and 75^th ^percentiles: $53,941; $136,878] v. $21,329 [25^th ^and 75^th ^percentiles: $11,403; $41,461]; p < 0.001). LOS and total hospital costs following the onset of Gram-negative sepsis were strongly correlated (Spearman's rank correlation coefficient = 0.947; p < 0.001).

When only hospital survivors were examined, subjects receiving prior antibiotic exposure had an unadjusted median increase of 7.0 days in LOS (16.0 days v. 9.0 days, p < 0.001). Similarly, when only hospital survivors were examined total median hospital costs were significantly greater for patients with prior antibiotic exposure compared to patients without prior antibiotic exposure (median values: $93,764 [25^th ^and 75^th ^percentiles: $52,892; $134,310] v. $21,369 [25^th ^and 75^th ^percentiles: $11,981; $39,760]; p < 0.001).

## Discussion

Our study suggests that prior antibiotic exposure was associated with greater LOS following the onset of Gram-negative bacteremia complicated by severe sepsis or septic shock. This observation was confirmed in an adjusted analysis of LOS and in a multivariate analysis controlling for potential confounding variables. Not surprisingly, other important determinants of prolonged LOS in the multivariate analysis included pre-infection onset hospital LOS, inappropriate antibiotic therapy, severity of illness, and the lungs as the source of infection.

A likely explanation for the association we observed between hospital LOS and prior antibiotic exposure is the greater occurrence of antimicrobial resistance among the causative pathogen(s) associated with sepsis among patients having received prior antibiotic therapy. We previously demonstrated that prior antibiotic exposure was associated with a greater incidence of infection attributed to Gram-negative bacteria that were either antibiotic-resistant or multi-drug resistant [17]. Prior antibiotic exposure was also associated with greater administration of inappropriate initial antimicrobial therapy which has been linked with excess mortality and longer lengths of stay in septic patients [3-7]. Shorr et al. evaluated the impact of a sepsis management protocol that emphasized identification of septic patients, aggressive fluid resuscitation, timely antibiotic administration, and appropriateness of antibiotics [6]. Use of the sepsis protocol was associated with lower hospital mortality and substantial institutional cost savings. The sepsis protocol significantly increased the use of appropriate initial antibiotic therapy, despite the initial use of potentially more costly antibiotic regimens, and represents a strategy for enhancing resource utilization while containing overall costs in hospitalized patients with sepsis [6].

The findings from our study are consistent with those observed by other investigators. Webster et al. recently described resistance trends in *Escherichia coli *and *Klebsiella pneumoniae *bloodstream infections in Oxfordshire, UK, over an 11 year period [22]. In their univariate analysis multidrug resistance (OR 2.77, 95% CI 1.23-6.22, p = 0.014), previous hospital admission (OR 1.27, 95% CI 1.01-1.60, p = 0.041) and isolation of *Klebsiella pneumoniae *(OR 1.53, 95% CI 1.05-2.22, p = 0.026) predicted a longer hospital LOS, as did age 7 years (OR 1.58, 95% CI 1.26-1.99, p < 0.0005). On multivariate analysis, multidrug resistance remained a strong independent predictor of increased LOS. Similarly, Lautenbach et al. examined the longitudinal trends in prevalence of imipenem-resistant *Pseudomonas aeruginosa *(IRPA) from 2 centers from 1989 through 2006 [23]. Isolation of IRPA was associated with longer hospital LOS after culture (p < .001) and greater hospital costs after culture (p < .001) than was isolation of an imipenem-susceptible strain. In multivariable analysis, IRPA infection or colonization remained an independent risk factor for both longer hospital LOS after culture and greater hospital costs after culture. Although these investigators did not specifically examine prior antibiotic exposure as a risk factor, antibiotic-resistant bacterial infection represents a good marker for having had such exposure occur [12,13].

Our study is unique in identifying prior antibiotic exposure as an independent risk factor for prolonged LOS among patients with Gram-negative bacteremia complicated by severe sepsis or septic shock. This observation suggests that clinicians should search for and identify the presence of prior antibiotic exposure as an important consideration when prescribing empiric antibiotic therapy to patients with severe sepsis or septic shock. The identification of prior antibiotic exposure should result in specific therapeutic interventions. For example, clinicians should avoid antibiotics or antibiotic classes to which the patient was previously exposed. Additionally, severity of illness appears to play an important role in determining which patients are at greatest risk for adverse outcomes from inappropriate antibiotic therapy [17]. Patients with shock or neutropenia, especially those with prior antibiotic exposure, may benefit the most from initial combination therapy that includes an aminoglycoside in order to minimize the occurrence of inappropriate initial antimicrobial therapy [24,25].

There are several important limitations of our study that should be noted. First, the study was performed at a single center and the results may not be generalizable to other institutions. However, the findings from other investigators corroborate the potential role of prior antibiotic exposure as an important determinant of outcome, including LOS, for patients with serious Gram-negative infections [5-7,24]. Second, the retrospective and observational nature of this study limits our ability to establish causality between prior antibiotic exposure and hospital LOS. The retrospective design also restricts our ability to identify all prior antibiotic exposure, especially outpatient exposure to antimicrobial agents. However, we are confident that the majority of all antibiotic exposure in the 90 days prior to severe sepsis onset was captured by our data base, especially as it captures cli9nical data from both inpatient and outpatient facilities. Third, our study may not have identified all of the factors contributing to length of stay in this patient population. Fourth, we did not evaluate the number of days preceding hospital admission when prior antibiotic exposure occurred nor did we assess the total number of days of prior antibiotic exposure. These could be other important variables that influence patient outcomes. Lastly, the presence of prior antimicrobial exposure may not be easily identified, especially in patients without easily accessible medical records describing recent outpatient and inpatient treatments. This is an important limitation for using the risk factor of prior antibiotic exposure in daily patient management decision making.

## Conclusions

In conclusion, we observed that prior antibiotic exposure is relatively common and associated with adverse outcomes including greater hospital LOS among patients with Gram-negative bacteremia complicated by severe sepsis or septic shock. This observation suggests that clinicians treating patients with suspected Gram-negative bacteremia or sepsis should attempt to identify whether prior antibiotic exposure occurred. In clinical situations where recent antibiotic exposure is likely, but details concerning prior antibiotic exposure are unknown, combination empiric therapy directed against Gram-negative bacteria would be reasonable to increase the likelihood of appropriate therapy, until susceptibility data become available. Given the importance of prior antibiotic exposure as a risk factor for antibiotic resistance, inappropriate therapy, and increased mortality, and the availability of electronic medical records at many hospitals, institutions should try to formalize an approach for identifying prior antibiotic exposure in patients with serious infections.

## Abbreviations

LOS: length of stay; ICD - 9 - CM: International Classification of Diseases, Ninth Revision, Clinical Modification; APACHE: Acute Physiology and Chronic Health Evaluation.

## Competing interests

Dr, Kollef's efforts were supported by the Barnes-Jewish Hospital Foundation. The authors have no other financial conflicts of interest or competing interests regarding this manuscript. Sharp & Dohme Corp. provided an unrestricted grant in support of this and other investigations. They had no role in the development or the conduct of this investigation.

## Authors' contributions

SM had full access to all of the data in the study and takes responsibility for the integrity of the data and the accuracy of the data analysis. SM contributed to the study conception and design, statistical analysis, and critical revision of the manuscript. MJ had full access to all of the data in the study and takes responsibility for the integrity of the data and the accuracy of the data analysis. MJ contributed to the study conception and design, acquisition of the data, statistical analysis, and drafting of the manuscript. RR contributed to acquisition of the data and critical revision for important intellectual content. MK had full access to all of the data in the study and takes responsibility for the integrity of the data and the accuracy of the data analysis. MK contributed to the study conception and design, statistical analysis, and critical revision of the manuscript. All authors read and approved the final manuscript

## Pre-publication history

The pre-publication history for this paper can be accessed here:

http://www.biomedcentral.com/1471-2334/12/56/prepub
